# Multidisciplinary Approach for a Rare Metastatic Low-Grade Endometrial Stromal Sarcoma to the Inferior Vena Cava and the Right Atrium

**DOI:** 10.7759/cureus.32807

**Published:** 2022-12-21

**Authors:** Hasan M Alswiket, Hawra M Aldar, Ryad F Alamry, Zahra S Albahrani, Maram A Alismail, Yasser A Elghoneimy

**Affiliations:** 1 College of Medicine, Imam Abdulrahman Bin Faisal University, Dammam, SAU; 2 Department of Cardiac Surgery, King Fahad University Hospital, Dammam, SAU

**Keywords:** pulmonary embolism, intracardiac tumor, ivc thrombus, endometrial stromal sarcoma

## Abstract

Endometrial stromal sarcoma (ESS) is a rare, malignant tumor of the endometrium. Low-grade endometrial stromal sarcoma (LG-ESS) is a less aggressive subtype of ESS that rarely metastasizes to the heart and large blood vessels. In the present study, we report a case of recurrent LG-ESS after treating the initial mass in the uterus six years ago in a 49-year-old female who presented with a four-month history of dyspnea and easy fatigability. Investigations revealed a right pulmonary embolism, suspicious right psoas muscle mass, and a large inferior vena cava (IVC) thrombus. One month later, she presented with multiple gastrointestinal symptoms and weight loss. Investigations then showed the development of a new right atrial mass, infra-diaphragmatic metastatic lymphadenopathy, progression of the presacral soft tissue component, invasion of the ileal bowel loop, and a tumoral thrombus in the IVC besides new metastatic lymphadenopathy and pulmonary metastasis. Therefore, a multidisciplinary team, which had a crucial role in this complicated case, decided to commence chemotherapy treatment. Such an unusual aggressive metastatic course of LG-ESS is limited in the literature; herein, we recognize a rarely documented disease.

## Introduction

Endometrial stromal sarcoma (ESS) is a very rare neoplasm that constitutes about 0.2% of all uterine malignancies and 7%-15% of uterine sarcomas [1,2]. Low-grade endometrial stromal sarcoma (LG-ESS), a subtype of ESS, is a malignant tumor composed of cells that resembles the proliferative phase of the endometrial stroma, showing permeative and infiltrative invasion into the myometrium or lymphovascular structures [3]. Generally, the prognosis is good; however, approximately 50% of patients with LG-ESS experience tumor recurrence, which typically occurs after a long period of latency [2]. ESS metastases rarely involve large blood vessels or the heart due to continuous myocardial contractility, rapid blood flow, and lymphatic drainage away from the heart [3,4]. On the other hand, most cases of LG-ESS metastasize to the pelvis, abdomen, and lungs [5]. Herein we report a case of a 49-year-old Saudi female with recurrent LG-ESS in the right psoas muscle with metastatic spread into the inferior vena cava (IVC) and the right atrium.

## Case presentation

We report a case of a 49-year-old Saudi female with a past medical history of diabetes and hypertension, who presented with a recurrence of a LG-ESS six years after initial management of the disease with pelvic radiotherapy, brachytherapy, and total hysterectomy including bilateral salpingo-oophorectomy. Recently, she presented with a four-month history of dyspnea on minimal effort and easy fatiguability. No significant findings were seen on her chest x-ray. Computed tomography (CT) of the chest and abdomen revealed right pulmonary embolism and right kidney hydronephrosis, which was managed by placement of a nephrostomy tube. In addition to these findings, there was an incidental detection of a suspicious right psoas muscle mass, measuring approximately 3.6X2.6cm, and a large IVC thrombus that did not extend into the right atrium. Furthermore, histological biopsy of the primary mass in the right psoas muscle revealed estrogen receptor-positive LG-ESS. Hence, she was started on anticoagulant (enoxaparin) and hormone replacement therapy (tamoxifen).

One month after starting enoxaparin and tamoxifen, she developed sudden, intermittent, progressive epigastric pain, episodes of vomiting, hematochezia, dark urine, and a weight loss of 4kg in one week. Therefore, CT scan of the chest, abdomen, and pelvis was repeated which revealed additional findings of a right atrial mass, infra-diaphragmatic metastatic lymphadenopathy, progression of the presacral soft tissue component, and invasion of the ileal bowel loop with borderline dilation of the proximal small bowel segment, suggestive of partial bowel obstruction. The patient was managed symptomatically with paracetamol and fentanyl for her epigastric pain, and metoclopramide was given for nausea and vomiting. Further, partial intestinal obstruction was managed via nasogastric tube (NGT) insertion. Also, tamoxifen was discontinued because it was contraindicated for the patient, and the mass continued growing despite the treatment.

**Figure 1:**
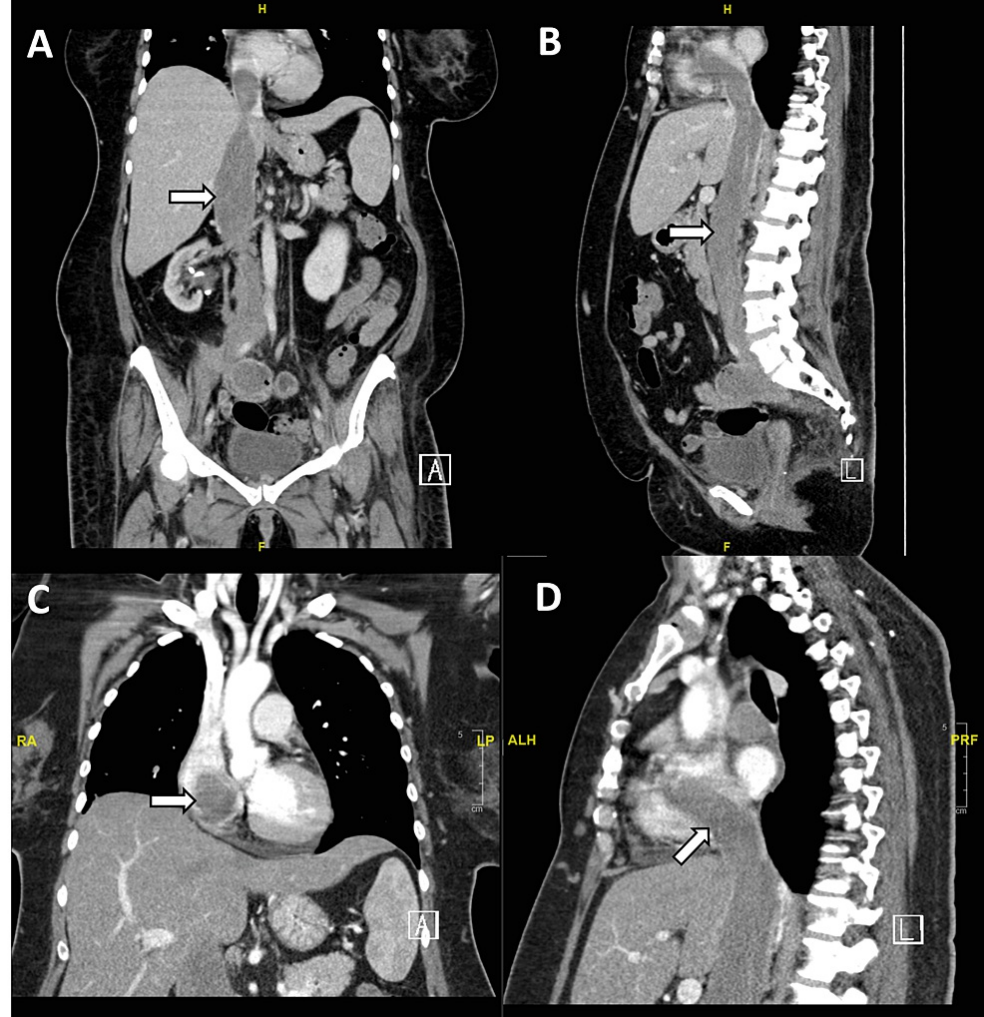
CT scan with contrast (coronal and sagittal views) of the chest, abdomen, and pelvis. CT scan views (A,B) demonstrate a soft tissue mass in the right psoas muscle with near total occlusion of the inferior vena cava by thrombus (white arrows in A,B), and (C,D) extension into the right atrium (white arrows in C,D).

**Figure 2:**
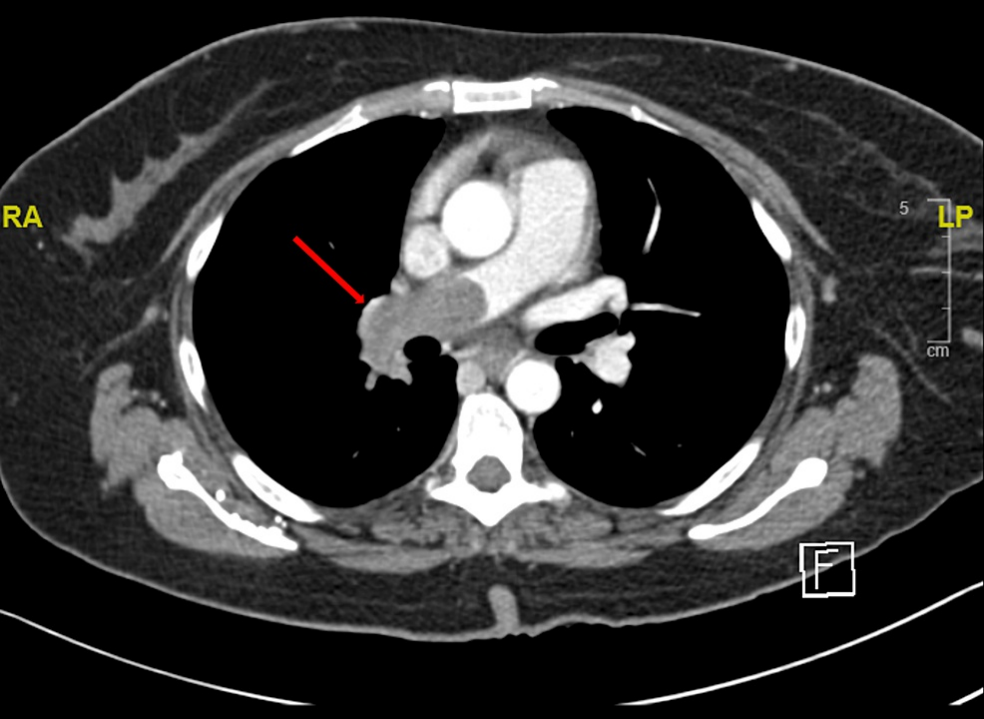
CT with contrast (axial view) of the chest showing pulmonary embolism (red arrow) of the right pulmonary artery.

Because the lesion was located in the IVC and extended into the right atrium, a cardiovascular surgery consultation was obtained, so she was transferred to our hospital. To reassess the mass progression, chest, abdominal, and pelvic CT scan was repeated two weeks after the previous CT scan, and it showed further interval progression of the primary mass that had markedly expanded the IVC with thrombus, extending into the right atrium, as well as the presence of a right pulmonary artery embolism (Figure 1, 2).

**Figure 3:**
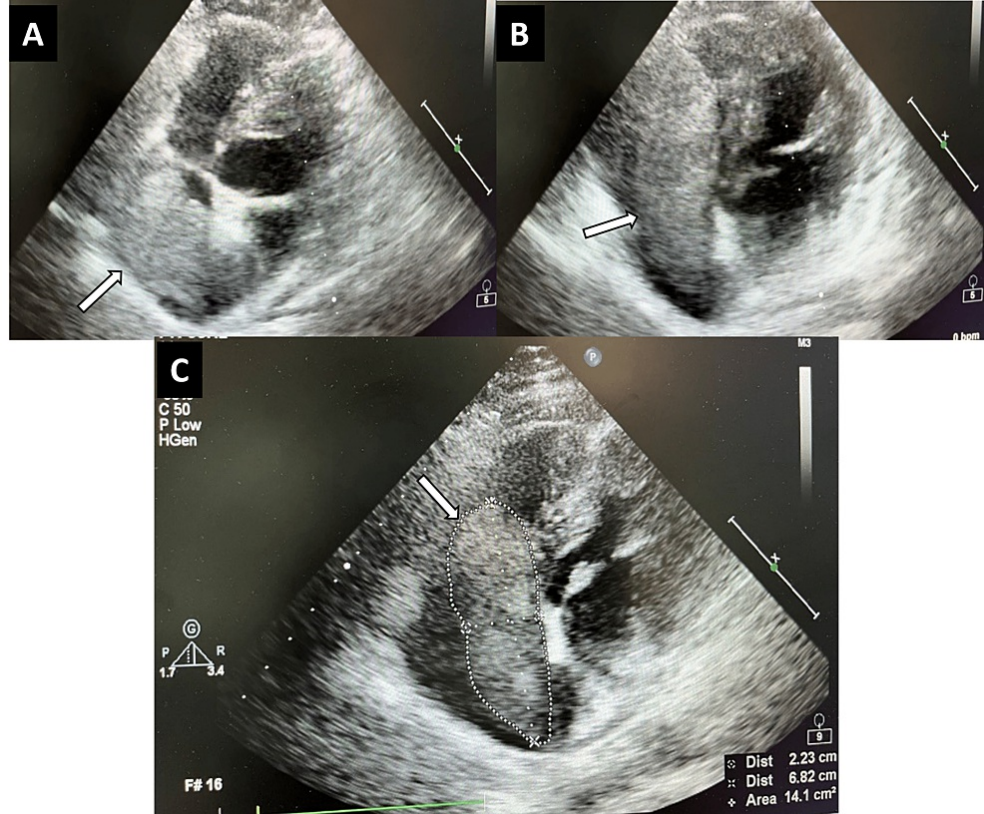
Transthoracic echocardiography showing a huge mass in the right atrium. (A) A rather large, highly mobile mass (white arrows in A,B) in the right atrium (B) moves towards the right ventricle with cardiac contractions. (C) Total area of the mass (white arrow/circled mass in C) is 14.1 cm^2^.

**Figure 4:**
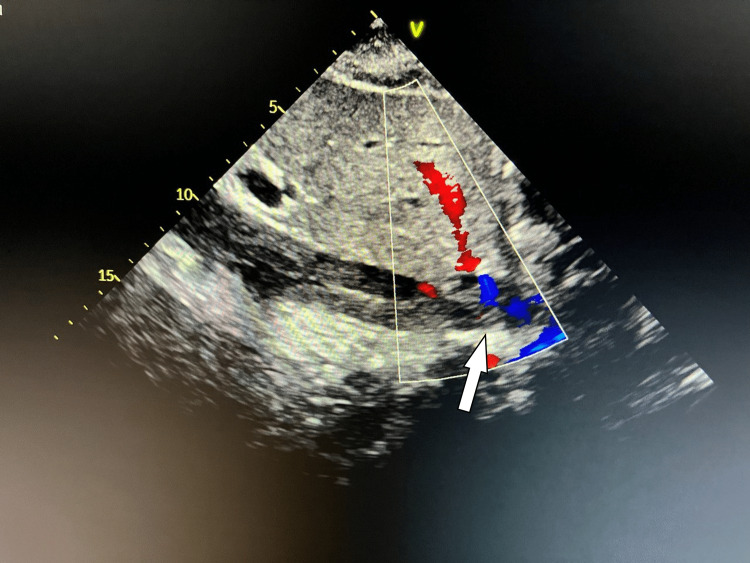
Color doppler echocardiography (ECHO) showing dilation and near total occlusion of the inferior vena cava (white arrow) with considerably reduced flow.

In transthoracic echocardiography (ECHO), a large, highly mobile mass in the right atrium which occupied approximately two-thirds of the chamber with a total area of 6.46cm^2^ was seen. To reevaluate the need for urgent surgical intervention to prevent unpredictable embolization, reassessment of the size and mobility of the right atrial mass was needed, so ECHO was repeated one week later and showed that the previously identified large mass in the right atrium had doubled in size with a total area of 14.1cm^2^, and appeared to be more mobile, prolapsing into the right ventricle during diastole (Figure 3). Additionally, color doppler echocardiography showed dilation and near-total occlusion of the IVC with considerably reduced blood flow (Figure 4).

**Figure 5:**
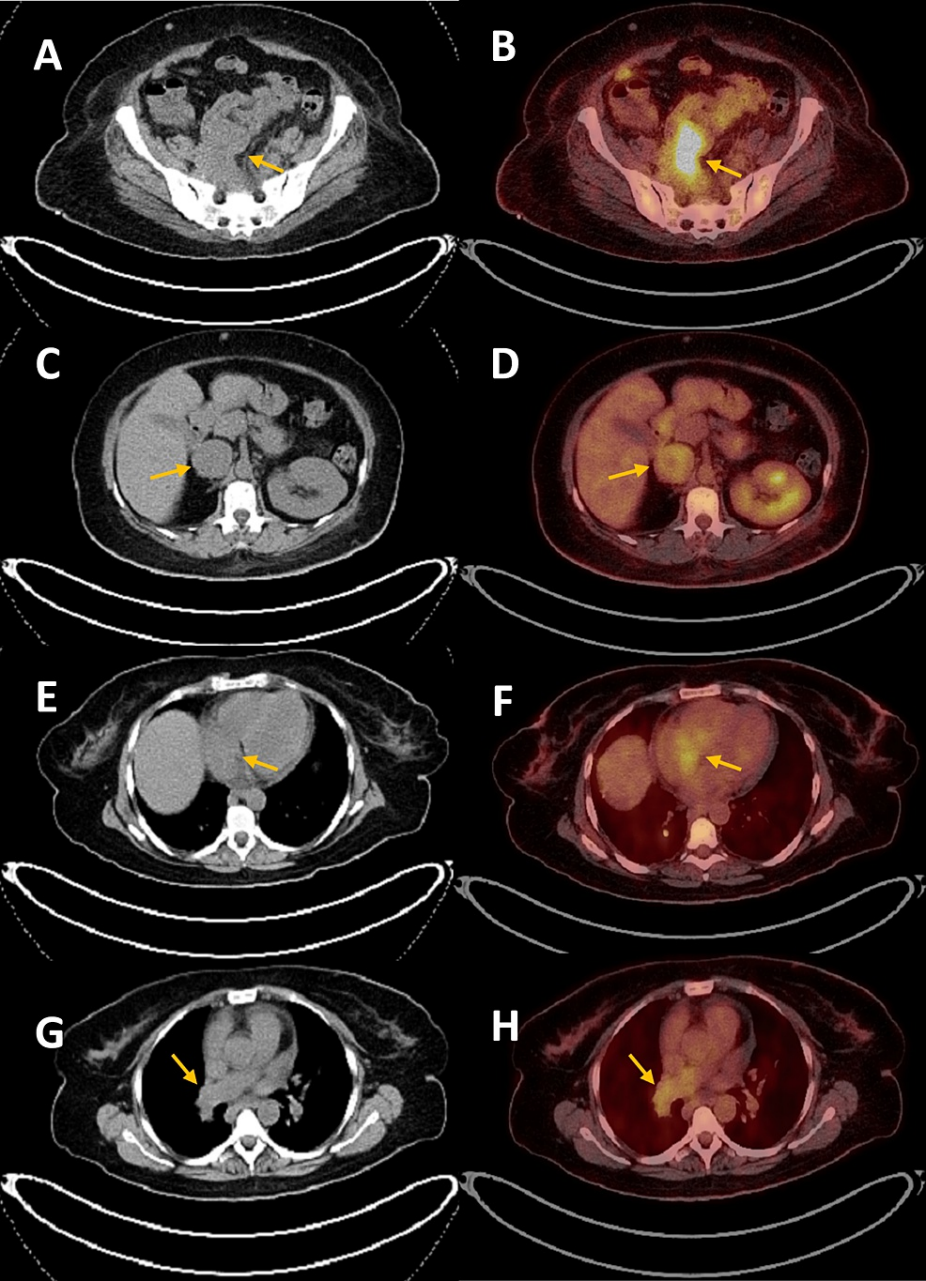
Axial 18F-FDG PET/CT without contrast of the whole body. Scans (A,B) reveal increased FDG activity of the irregularly shaped soft tissue mass (orange arrows in A,B) invading the right psoas muscle, which is inseparable from the adjacent small bowel loops at the pelvic inlet. The dimensions of the mass are 6.0 cm x 3.1 cm (SUVmax 8.3), which previously was measured at 3.6 cm x 2.6 cm (SUVmax 2.5). Also, it shows (C,D) inferior vena cava enlargement (orange arrows in C,D) with increased FDG activity, (E,F) high FDG uptake of the right atrial mass (orange arrows in E,F), and (G,H) high FDG uptake of the right pulmonary embolism (orange arrows in G,H). 18F-FDG PET/CT: Flourine-18 fluorodeoxyglucose positron emission tomography/computed tomography

**Figure 6:**
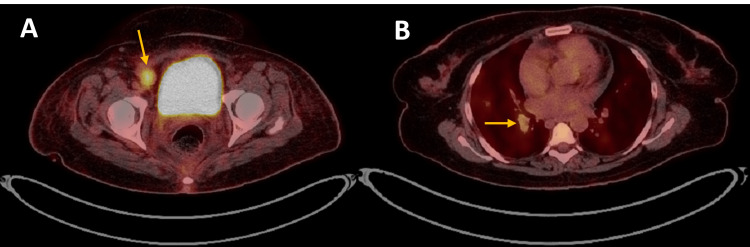
Axial 18F-FDG PET/CT (Chest and pelvis sections) There is (A) FDG avid right common iliac and right external iliac lymphadenopathy (orange arrow in A) that measures 2.3 cm^2^ (SUVmax 6.3), and (B) small nodular opacities in the right middle lobe. The largest lung nodule (orange arrow in B) measures 1.4 cm^2^ (SUVmax 1.8). 18F-FDG PET/CT: Flourine-18 fluorodeoxyglucose positron emission tomography/computed tomography

Flourine-18 fluorodeoxyglucose positron emission tomography/computed tomography (18F-FDG PET/CT) whole body scan was performed together with a low dose, non-contract CT scan. The scan detected an interval progression in size together with increased FDG activity of the previously seen irregularly shaped soft tissue mass that was anterior to the right psoas muscle. Likewise, the previously seen IVC thrombosis exhibited increased FDG uptake, reflecting that the thrombus is probably tumoral. Furthermore, there was an abnormal uptake of FDG into the right atrial mass and the pulmonary embolus (Figure 5). Lastly, FDG avid metastatic lymphadenopathy and small nodular opacities in the right middle lung lobe were detected, which was suspicious for pulmonary metastasis (Figure 6). Due to the aggressive local invasion of the primary tumor, and its distal metastasis, the possibility for an operative intervention was rejected by oncological surgeons, and chemotherapy was recommended. Based on that, a decision was made by the multidisciplinary team to recommend chemotherapy for this stage of disease. Currently, the patient is stable, undergoing chemotherapy (gemcitabine/docetaxel combination therapy), receiving enoxaparin for three months, and waiting for any improvement in her condition.

## Discussion

Endometrial stromal sarcoma is a rare, but aggressive uterine sarcoma subtype that has a mesenchymal origin. It could be a low-grade or high-grade ESS depending on its differentiation and its degree of invasion into the myometrium [6,7]. A low-grade ESS tends to invade locally or into lymphovascular structures [6]. It is unusual for a low-grade malignancy to metastasize; however, LG-ESS has a metastatic potential that usually occurs in the pelvis, abdomen, and lungs [8,9]. Metastatic spread into the IVC is rare compared to renal cell carcinoma which is the most common malignancy seen to extend into the IVC [4]. Potential risk factors for ESS are not evident; however, some studies have shown a relationship to tamoxifen [10]. Typically, patients present with menorrhagia, but many remain asymptomatic [11]. The ideal management for ESS is to perform a total hysterectomy and bilateral salpingo-oophorectomy. Although surgery is usually curative for LG-ESS, a recurrence is still possible, even after 25 years [6,12].

Regarding the histopathological characteristics, LG-ESS cells are uniform and resemble endometrial stroma in its proliferative phase. Under the microscope, stromal cells are densely uniform with some mitotic figures and mild nuclear atypia [13]. Hormonally, most LG-ESS tumors are estrogen and progesterone receptor-positive [5].

The role of surgical intervention for uterine ESS is also present for extrauterine ESS [14], and advanced presentation of LG-ESS [2]. Also, endocrine therapy has shown its efficacy in the treatment of recurrent LG-ESS [15]. Usually, chemotherapy administration is for those with progressive LG-ESS despite hormonal therapy [16]. In one of the cases similar to our patient, postoperative administration of carboplatin and paclitaxel lead to a better prognosis for the patient [17]. The choice of one or more of these modalities is dependent on the presentation.

This patient presented with a recurrent LG-ESS in the right psoas muscle that had extended into the entire IVC and into the right atrium. The mass in the right atrium rapidly increased in size and together with its high mobility nature, embolization is a possibility that could occlude the main pulmonary artery causing immediate sudden death. Consequentially, the patient was referred to our hospital for surgical resection of the mass in the right atrium as it has a high risk of embolization. Further, after resecting the mass in the right atrium and the embolus in the right pulmonary artery, the patient would require an additional surgical procedure to resect the primary tumor. Due to the fact that the primary tumor appears to be aggressively invasive with distal metastases to other organs, the oncology surgeons agreed that a curative resection should be avoided and the use of chemotherapy was the most logical therapeutic option. Under those circumstances, resecting the right atrial mass and the right pulmonary artery embolus without removing the primary tumor and its extension into the IVC carries a high recurrence rate making surgical intervention irrational. The patient is already on enoxaparin for more than three months, but it is not showing any effect, and this could be due to the fact that the thrombus in the IVC is not a pure thrombus but a tumoral thrombus which is explained by its increased FDG uptake in the PET CT scan. AngioVac system has been effective in multiple cases with total iliocaval thrombosis; however, those cases were pure thrombosis [18]. On the other hand, it was ineffective in a case similar to our patient due to the adherent nature of the tumoral thrombosis [19]. Cavatomy and right atriotomy has been effective in multiple cases with IVC tumoral thrombus extending into the right atrium, right ventricle, and right pulmonary artery using cardiopulmonary bypass [2]. Nevertheless, it is not indicated for our patient because the primary tumor in the psoas muscle is unresectable, so the risks overweight the benefits, and the recurrence rate is high. In such a relatively stable patient with an advanced stage of malignancy, while all surgical and minimally invasive options were rejected, and since hormone replacement therapy (tamoxifen) was contraindicated, palliative chemotherapy seems to be the only available management. In addition to chemotherapy, symptomatic treatment might be indicated according to the patient’s complaint.

The last resort for our patient was to start palliative chemotherapy as soon as possible to prevent further growth and reduce the tumor size in an attempt to improve the symptoms and prevent the development of further complications. Depending upon the response from the chemotherapy, future surgical involvement could be an option. However, a decision for surgical intervention could be considered for life-saving situations like when the primary tumor grows aggressively causing bowel obstruction, or when the risk of embolic showering increases due to the progressive growth of the intracardiac tumor. Under those circumstances, the right atrial mass resection would not change the long-term outcome of the patient, but it would have an immediate life-saving result. 

This difficult case proves that even though low-grade sarcomas have a good prognosis and are less likely to metastasize [20], LG-ESS has a metastatic potential, and could complicate the case, so it should never be neglected.

## Conclusions

Low-grade endometrial stromal sarcoma describes an uncommon subtype of an ESS that very rarely metastasizes to the heart. Its tendency to infiltrate the myometrium and lymphovascular structures, metastasize, and re-occurrence accounts for its unique nature. Though it is considered an aggressive malignancy, it usually has a good prognosis. Therefore, early diagnosis and timely management may contribute to improved survival. We report a case of recurrent LG-ESS with uncommon metastatic locations into the IVC, right atrium, pulmonary parenchyma, and adjacent lymph nodes. Such a case requires complicated multidisciplinary cooperation to achieve the desired outcome.
